# Supplementary material for: Long, Noncoding RNA SRA Induces Apoptosis of β-Cells by Promoting the IRAK1/LDHA/Lactate Pathway
Source: Int J Mol Sci. 2021 Feb 9;22(4):1720. doi: 10.3390/ijms22041720 (PMC7914996; doi:10.3390/ijms22041720)
Supplement: Supplementary file 1 [file ijms-22-01720-s001.pdf]

Supplemental Information

Table S1. LncRNAs involved in metabolism. (Related to Figure 1)

LncRNAs	Metabolism-related enzyme	Tumor/cell types	References
SRA	PPARA, PPARG, FABP4; ATGL	Liver, Hepatocytes	1, 2
HAND2-AS1	GLUT1, GLUT3	Osteosarcoma	3
HOTAIR	GLUT1	Hepatocellular carcinoma	4
SNHG3	PFK, PKM, CS, IDH, OGDH	Ovarian cancer	5
TUG1	HK2	Hepatocellular carcinoma, Osteosarcoma	6, 7
CRYBG3	LDHA	Lung cancer	8
PCGEM1	G6PD	Prostate cancer	9
GLCC1	LDHA	Colorectal cancer	10
YIYA	PFKFB3	Breast cancer	11
PVT1	HK2	Gallbladder cancer	12

1. Liu, S. *et al.* SRA Gene Knockout Protects against Diet-induced Obesity and Improves Glucose Tolerance. *J. Biol. Chem.* **289**, 13000–13009 (2014).
2. Chen, G. *et al.* LncRNA SRA promotes hepatic steatosis through repressing the expression of adipose triglyceride lipase (ATGL). *Sci Rep* **6**, 35531 (2016).
3. Kang, Y. *et al.* Energy stress-induced lncRNA HAND2-AS1 represses. 12.
4. Wei, S. *et al.* Promotion of glycolysis by HOTAIR through GLUT1 upregulation via mTOR signaling. *Oncology Reports* **38**, 1902–1908 (2017).
5. Li, N., Zhan, X. & Zhan, X. The lncRNA SNHG3 regulates energy metabolism of ovarian cancer by an analysis of mitochondrial proteomes. *Gynecologic Oncology* **150**, 343–354 (2018).
6. Lin, Y.-H. *et al.* Taurine up-regulated gene 1 functions as a master regulator to coordinate glycolysis and metastasis in hepatocellular carcinoma: Lin et al. *Hepatology* **67**, 188–203 (2018).

7. Han, X., Yang, Y., Sun, Y., Qin, L. & Yang, Y. LncRNA TUG1 affects cell viability by regulating glycolysis in osteosarcoma cells. *Gene* **674**, 87–92 (2018).
8. Chen, H. *et al.* Long non-coding RNA CRYBG3 regulates glycolysis of lung cancer cells by interacting with lactate dehydrogenase A. *J. Cancer* **9**, 2580–2588 (2018).
9. Hung, C.-L. *et al.* A long noncoding RNA connects c-Myc to tumor metabolism. *Proc Natl Acad Sci USA* **111**, 18697–18702 (2014).
10. Tang, J. *et al.* LncRNA GLCC1 promotes colorectal carcinogenesis and glucose metabolism by stabilizing c-Myc. *Nat Commun* **10**, 3499 (2019).
11. Xing, Z. *et al.* Expression of Long Noncoding RNA *YIYA* Promotes Glycolysis in Breast Cancer. *Cancer Res* **78**, 4524–4532 (2018).
12. Chen, J. *et al.* Long non-coding RNA PVT1 promotes tumor progression by regulating the miR-143/HK2 axis in gallbladder cancer. *Mol Cancer* **18**, 33 (2019).

Table S2. Prediction of highly conserved miRNAs related to regulation of lncRNA SRA1*. (Related to Figure 1)

MicroRNA family	Seed position	Conservation		
		Primates	Mammals	Other vertebrates
miR-146ac/146b-5p	chr5:139936817	78%	70%	31%
miR-148ab-3p/152	chr5:139930781	67%	61%	0%
miR-203	chr5:139930110	78%	52%	0%
miR-103a/107/107ab	chr5:139930711	78%	52%	0%
miR-216a	chr5:139929757	67%	30%	0%
miR-216b/216b-5p	chr5:139929757	67%	30%	0%
miR-124/124ab/506	chr5:139937248	67%	30%	0%
miR-29abcd	chr5:139930820	67%	17%	0%
miR-208ab/208ab-3p	chr5:139931159	67%	4%	0%
miR-499-5p	chr5:139931159	67%	4%	0%

*, Bioinformatic information was obtained from miRcode

(http://www.mircode.org/)

Table S3. The primer information for quantitative PCR

Primer names	Primer sequences (5′–3′)
mLDHA-F	AAC TGG GCA CTG ACG CAG AC
mLDHA-R	GCC AAT GGC CCA GGA TGT GT
mHK2-F	GGC AGT GGA ACC CAG CTG TT
mHK2-R	CCC AGC GGG AGC TTC TTC TC
SRA-F	CCT ATT TGC ACT GTA TCA CCC
SRA-R	CCC CAA TCT CAG TAA TCT GG
mPFKL-F	CTC AGC CCT GCA CCG CAT TA
mPFKL-R	GAA GCC AGG GCA GAC ACC AG
mMDH2-F	GCT CGA GTC AAC GTG CCT GT
mMDH2-R	GCA GAA CCT GCT CCA GCC TT
mGOT2-F	ACC CAG CTG GTC TCC AAC CT
mGOT2-R	CCA CGG AGA TTC GGC CAT CC
mGPT2-F	TGG AGG CAG CTC AGT CCC AT
mGPT2-R	GGC ACG ACA CAG ATG CCA GT
mGLUD1-F	CGC CCT GCA AGG GAG GTA TC
mGLUD1-R	CGC CTG CTT TAG CAC CTC CA
mG6PD-F	CCC AGG TGT GTG GGA TCC TG
mG6PD-R	CCC GGA ACA GCC ACC AGA TG
mACLY-F	AGT GCC ACC TCC AAC AGT GC
mACLY-R	CCT GCC CTC GCT CAT CAC AG
mTKT-F	CCG AGC AAC CAA AGG CAG GA
mTKT-R	CCC ACT ACG GCA GCA GAC AC
mTADO1-F	CAG CCC AGA TGC CTG CCT AC
mTADO1-R	CTT GAG GCC CAC CCA GCT TC
mFH-F	GGC CGC AGA TGA GGT AGC TG
mFH-R	GGG TGC ACA GGC TTC TTG CT
mSDHA-F	GAG CCT GTG CCC TGA GCA TT
mSDHA-R	CAC GGA ACA CTG CAG CAT GG
mPDHA-F	CTG CTG CGC TCC ATG AGG AA
mPDHA-R	ACG GGA AGC AAC CAG CAC TC
mPC-F	GGC ACA GTG GAC ACC CAG TT
mPC-R	AGC TGG AGG TGG GCC TAT GG
mGLS1-F	TGC CCT CCG AAG GTT TGC TC
mGLS1-R	CTC TGC TGC TGC GAC ATG GA

mCPT1A-F	CAC CAC TGG CCG CAT GTC AA
mCPT1A-R	GAG CAG CAC CTT CAG CGA GT
mSLC16A1-F	GCT GGT GGT TGT CTG TCT GG
mSLC16A1-R	GCA AGC CCA AGA CCT CCA AT
mSLC16A3-F	TCC ATC CTG CTG GCT ATG CT
mSLC16A3-R	GAC CCA AGC CAG TGA TGA CC
mGLUT1-F	CGT GGC CAT CTT CTC TGT CG
mGLUT1-R	CCA TAA GCA CAG CAG CCA CA
mGLUT4-F	GCT GTC GCT GGT TTC TCC AA
mGLUT4-R	GGA CCC ATA GCA TCC GCA AC
hIL2RA-F	GCT CTG CCA CTC GGA ACA CA
hIL2RA-R	CCT GCA GTG ACC TGG AAG GC
hTNFRSF18-F	ACG AAG GCC ACT GCA AAC CT
hTNFRSF18-R	GCA CAC AGC GTT GTG GGT CT
hFOXP3-F	GAC AGC ACC CTT TCG GCT GT
hFOXP3-R	GCC TGG CAG TGC TTG AGG AA
hIKZF2-F	CAG CGA GGT GGC TGA CAA CA
hIKZF2-R	GCG TTC ACC ATT CGG AAG CC
hIKZF4-F	CCA ATG GCA AGC TCA AGT GT
hIKZF4-R	CCT TTC ACC AGT GTG ACT GC

m: Mouse; h: Human
